# Session Availability as a Result of Prior Injury Impacts the Risk of Subsequent Non-contact Lower Limb Injury in Elite Male Australian Footballers

**DOI:** 10.3389/fphys.2019.00737

**Published:** 2019-06-14

**Authors:** Joshua D. Ruddy, Samuel Pietsch, Nirav Maniar, Stuart J. Cormack, Ryan G. Timmins, Morgan D. Williams, David L. Carey, David A. Opar

**Affiliations:** ^1^School of Behavioural and Health Sciences, Australian Catholic University, Melbourne, VIC, Australia; ^2^Melbourne Football Club, Melbourne, VIC, Australia; ^3^School of Health, Sport and Professional Practice, Faculty of Life Sciences and Education, University of South Wales, Wales, United Kingdom; ^4^La Trobe Sport and Exercise Medicine Research Centre, College of Science, Health and Engineering, La Trobe University, Melbourne, VIC, Australia

**Keywords:** injury risk, prior injury, Australian football, logistic regression, injury prevenition

## Abstract

Prior injury is a commonly identified risk factor for subsequent injury. However, a binary approach to classifying prior injury (i.e., yes/no) is commonly implemented and may constrain scientific findings, as it is possible that variations in the amount of time lost due to an injury will impact subsequent injury risk to differing degrees. Accordingly, this study investigated whether session availability, a surrogate marker of prior injury, influenced the risk of subsequent non-contact lower limb injury in Australian footballers. Data were collected from 62 male elite Australian footballers throughout the 2015, 2016, and 2017 Australian Football League seasons. Each athlete’s participation status (i.e., full or missed/modified) and any injuries that occurred during training sessions/matches were recorded. As the focus of the current study was prior injury, any training sessions/matches that were missed due to reasons other than an injury (e.g., load management, illness and personal reasons) were removed from the data prior to all analyses. For every Monday during the in-season periods, session availability (%) in the prior 7, 14, 21, 28, 35, 42, 49, 56, 63, 70, 77, and 84 days was determined as the number of training sessions/matches fully completed (injury free) relative to the number of training sessions/matches possible in each window. Each variable was modeled using logistic regression to determine its impact on subsequent injury risk. Throughout the study period, 173 non-contact lower limb injuries that resulted in at least one missed/modified training session or match during the in-season periods occurred. Greater availability in the prior 7 days increased injury probabilities by up to 4.4%. The impact of session availability on subsequent injury risk diminished with expanding windows (i.e., availability in the prior 14 days through to the prior 84 days). Lesser availability in the prior 84 days increased injury probabilities by up to 14.1%, only when coupled with greater availability in the prior 7 days. Session availability may provide an informative marker of the impact of prior injury on subsequent injury risk and can be used by coaches and clinicians to guide the progression of training, particularly for athletes that are returning from long periods of injury.

## Introduction

Since 1992, an average of 92.5 injuries per 100 athletes have occurred in the Australian Football League (AFL) (Australian Football League, 2017). The overall incidence of injuries has not declined in recent years and the injuries that have occurred over this period have resulted in over 50,000 missed AFL matches (Orchard et al., 2013; Australian Football League, 2017). Injuries can impose a significant Financial burden on individual players and their clubs (Hickey et al., 2014) and can also negatively impact team and individual performances (Verrall et al., 2006; Drew et al., 2017), as well as physical and psychological wellness (Rozen and De L Horne, 2007). Given the toll these injuries can have, a large body of research has attempted to identify factors that increase or decrease the risk of injury (Orchard, 2001; Gabbe et al., 2004; Opar et al., 2012). The most commonly identified risk factor for subsequent injury is a history of injury (Orchard, 2001; Finch et al., 2017). As per previous work (Finch et al., 2017), a subsequent injury can be defined as any injury that occurs after an index (or first) injury. Accordingly, all recurrent injuries (i.e., the same pathology) are subsequent injuries. However, other subsequent injuries may not be related to the initial injury. Considering these definitions, prior lower limb strain injury has been associated with an increased risk of recurrent injury (Orchard, 2001). Additionally, prior injuries of all types have been linked to an increased risk of subsequent injuries (Finch et al., 2017).

Research typically focuses on modifiable risk factors, which can be targeted to reduce the risk of future injury (Bahr and Holme, 2003). However, significant interactions between prior injury and modifiable risk factors have been observed. For example, hamstring strength and fascicle lengths have been shown to moderate the impact of prior hamstring injury on the risk of subsequent hamstring injury (Opar et al., 2015; Timmins et al., 2016). Although prior injury is typically considered to be non-modifiable, it is important to consider (and quantify) how prior injury affects the risk of subsequent injury, as this can better inform practitioners when targeting modifiable factors to mitigate the risk of injury for specific athletes.

Traditionally, research has taken a binary approach to classifying injury history (Orchard, 2001; Arnason et al., 2004; Gabbe et al., 2006). Athletes are classified as either having or not having a prior injury, allowing the risk of subsequent injury to be compared for athletes with and without a prior injury. However, injuries can vary in severity and it is possible that variations in the amount of time lost due to an injury will impact the risk of subsequent injury to differing degrees. Accordingly, treating prior injury as a binary variable may not be the most informative approach. Prior injuries likely result in physiological deficits [i.e., reduced strength, reduced range of motion, altered muscle structure (Orchard et al., 2005)] that directly influence the risk of future injuries (Finch et al., 2017). However, it has been suggested that subsequent injury risk may also be influenced by the amount of training/competition missed during an injury layoff (Windt et al., 2016; Finch et al., 2017). Furthermore, it has been reported that greater levels of pre-season participation (i.e., completing more training sessions) decrease the odds of sustaining an injury during the in-season period for elite rugby league athletes (Windt et al., 2016). As such, it could be hypothesized that a greater number of training sessions and matches missed due to injury increases the risk of a subsequent injury occurring. No research, however, has investigated whether the amount of training/competition missed due to injury influences the risk of subsequent injury in elite athletes. Accordingly, the aim of the current study was to explore whether session availability, a surrogate measure of the amount of missed training sessions/matches due to prior injury, influenced the risk of subsequent injury in elite Australian footballers.

## Materials and Methods

### Study Design and Participants

This cohort study investigated the impact of session availability (as a surrogate marker of prior injury) on the risk of subsequent injury. Data were collected during the 2015, 2016, and 2017 AFL seasons (November 2014 to September 2017) and obtained by the research team. These data were collected from one team competing in the AFL. All athletes contracted to the team had their data included in this study (i.e., no athletes were excluded). Demographic data were recorded at the beginning of each season and reported to the research team. For every training session and match during the study period, each athlete’s participation status (i.e., full or missed/modified) and any injuries that occurred were recorded and reported to the research team. This study was approved by the Australian Catholic University Human Research Ethics Committee (approval number: 2018-26WN). The AFL team that participated in this research provided these data in a non-identified format to the research team without requiring the individual athletes to provide consent, as per the approved ethics application (approval number: 2018-26WN).

### Demographic and Injury Data

Demographic data were collected at the beginning of each season. These included date of birth, stature (cm), mass (kg), years of AFL experience, primary playing position [forward, back, midfield, or ruck (Ruddy et al., 2016)] and the number of games played in the prior season. For every field/skills training session and match during the study period, each athlete’s level of participation was reported as either full or missed/modified. If a training session or match was missed/modified, a reason was provided. If the reason for a missed/modified training session or match was an injury, defined as any physical complaint sustained by an athlete, excluding illness, details of the injury were recorded and provided. These details included the following:

• Location and type (e.g., hamstring strain, quadriceps contusion, navicular bone stress)
• Contact or non-contact (i.e., was contact with another athlete involved)
• First time, subsequent or recurrent (determined retrospectively according to Finch et al., 2017)
• Time until return to full participation

The aforementioned data were collected daily during the 2015, 2016, and 2017 AFL seasons by the team physiotherapist (SP) and were provided to the research team retrospectively.

### Data Analysis

As the focus of the current study was prior injury, any training sessions or matches that were missed due to any reasons other than an injury (e.g., load management, illness, and personal reasons) were removed from the data prior to all analyses. The proportion of training sessions and matches that were censored for these reasons can be found in Supplementary Material S1. For every Monday during the in-season periods, the number of training sessions and matches that were missed/modified due to injury in the prior 7, 14, 21, 28, 35, 42, 49, 56, 63, 70, 77, and 84 days was determined for each athlete. The number of full training sessions and matches that each athlete could have conceivably completed (if injury free) was also determined for the same retrospective windows. Session availability (%) for each retrospective window was then determined as the number of training sessions and matches fully completed (injury free) relative to the number of training sessions and matches possible for each athlete. For example, 100% session availability in the prior 7 days indicates that Athlete A was injury free and completed all training sessions and matches possible for them over the last week. Equally, 25% session availability in the prior 84 days indicates that Athlete B, due to injury, was unable to complete three quarters of training sessions and matches possible for them over the last 3 months. Non-contact lower limb injuries that occurred during the following 7 days (inclusive of the index day, i.e., Monday to Sunday) were identified as subsequent injuries.

As all injury types have been associated with an increased risk of subsequent injury (Finch et al., 2017), any type of injury (i.e., all contact, non-contact, upper limb and lower limb injuries), given it resulted in at least one missed/modified training session or match, was considered when calculating session availability. However, only non-contact lower limb injuries were considered as subsequent injuries (Colby et al., 2018). The risk of sustaining a contact injury is unlikely to be influenced by intrinsic or predisposing risk factors and as such, contact injuries are typically viewed as unavoidable (Gabbett, 2010). Any weeks during which an athlete did not train or play (i.e., was not exposed to the risk of subsequent injury) were not considered when identifying subsequent injury risk, but were still considered when calculating session availability (i.e., no training sessions or matches = 0% session availability). Given the impact that in-season injuries have compared to pre-season injuries in regards to missed matches (Orchard et al., 2013) and performance (Drew et al., 2017), the current study focused on identifying in-season injury risk. Pre-season data, however, were still considered when calculating session availability. It should also be noted that athletes who completed structured rehabilitation sessions were considered injured and unavailable for full (field/skills) training or matches, as per previous research (Ekstrand, 2013). A visual depiction of these data analysis steps can be found in Supplementary Material S2 and may assist the reader in better understanding the methods implemented.

### Statistical Analyses

Each individual variable was modeled using logistic regression (Supplementary Material S3). The coefficient for each variable was extracted and expressed as an odds ratio. An odds ratio of less than 1 indicates the factor by which the odds of a subsequent injury occurring are decreased with a one unit increase in the predictor variable (e.g., a 1% increase in session availability) (Peng et al., 2002). Conversely, an odds ratio of greater than 1 indicates the factor by which the odds of a subsequent injury occurring are increased with a one unit increase in the predictor variable (Peng et al., 2002). Probabilities are more intuitive than odds and are generally preferred by practitioners. However, probabilities are limited to values between zero and one (or 0% and 100%), whereas odds can range from zero to infinity. Logistic regression assumes a linear relationship between a predictor and the log odds of the outcome. Therefore, an odds ratio can be used to express the constant effect a one unit increase in the predictor has on the odds of the outcome. However, the effect a one unit increase in the predictor has on the probability of the outcome will not be uniform throughout the range of the predictor. Alternatively, logistic regression can be used to calculate the probability of the outcome at fixed values of the predictor.

A coefficient was determined statistically significant if the 95% confidence intervals (CIs) did not include a value of 1. The models were then used to estimate the probability of subsequent injury at each value between the minimum and maximum of each statistically significant variable. The equations used to estimate the probability of injury for all analyses can be found in Supplementary Material S3. The individual interactions of session availability in all windows by age and by games played in the prior season were explored. The interaction of session availability in the prior 7 days by session availability in the prior 84 days was explored, as these two variables, out of all the session availability variables, had the lowest correlation (Supplementary Material S4). Low levels of collinearity are important when modeling interactions using logistic regression (Dormann et al., 2013). The coefficient of the interaction of two variables can be difficult to interpret compared to a univariable coefficient. When expressed as an odds ratio, the interaction coefficient of variables A by B (assuming they are continuous) indicates the factor by which a one unit increase in A affects the odds of a subsequent injury occurring for every one unit increase in B (Ai and Norton, 2003). All data and statistical analyses were completed using R statistical programming language (R Core Team, 2013).

## Results

### Cohort and Subsequent Injury Details

**FIGURE 1:**
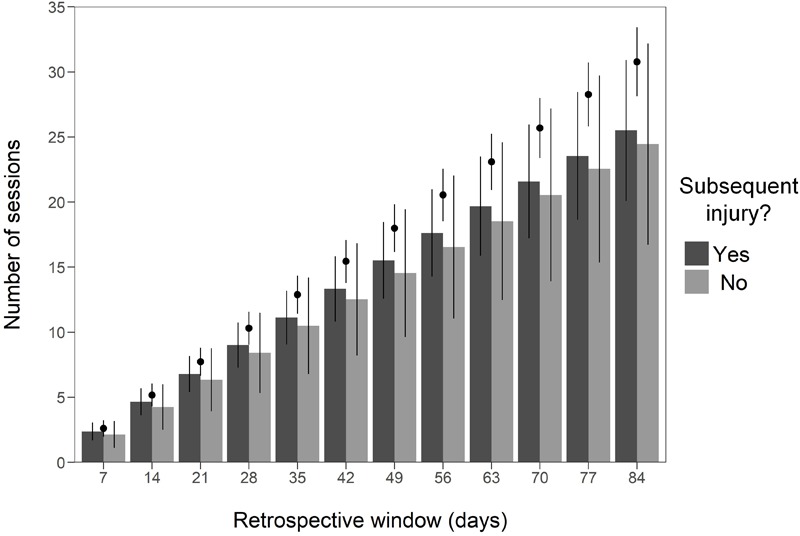
The average number of training sessions and matches fully completed ( ± standard deviation) in each retrospective window for weeks during which a subsequent injury did and did not occur. The black points represent the average amount of training sessions/matches that were possible ( ± standard deviation) in each retrospective window (i.e., the average number of training sessions/matches required to be fully completed to constitute 100% session availability).

Sixty-two male elite Australian footballers (age 23.7 ± 3.4 years, stature 187.8 ± 7.2 cm, mass 88.8 ± 8.0 kg, AFL playing experience 4.2 ± 3.4 years) from one team competing in the AFL provided data for this study over three seasons. Of these athletes, 18 participated across one season, 17 across two seasons, and 27 across all three seasons. Collectively, over the course of the study period, these athletes provided 3,369 weekly in-season observations. Throughout the study period, 173 non-contact lower limb injuries that resulted in at least one missed/modified training session or match during the in-season periods occurred. Of these injuries, 38% occurred during the 2015 season, 29% during the 2016 season, and 33% during the 2017 season. The median number of sessions and matches missed by individual athletes due to these injuries was 1 (interquartile range = 2) and 0 (interquartile range = 1), respectively. These injuries were sustained by 54 individual athletes (age 24.2 ± 3.4 years, stature 188.2 ± 7.4 cm, mass 89.2 ± 8.2 kg, AFL playing experience 4.4 ± 3.4 years). Eight athletes (age 21.2 ± 1.8 years, stature 187.2 ± 5.6 cm, mass 87.7 ± 6.1 kg, AFL playing experience 2.2 ± 2.3 years) did not sustain a non-contact lower limb injuries that resulted in at least one missed/modified training session or match during the in-season periods. The incidence and prevalence of injuries by location for each individual season can be found in Supplementary Material S5. Figure 1 illustrates the average number of training sessions/matches fully completed ( ± standard deviation) in each retrospective window for weeks during which a subsequent injury did and did not occur. Figure 1 also illustrates the average amount of training sessions/matches that were possible ( ± standard deviation) in each retrospective window (i.e., the average number of training sessions/matches required to be fully completed to constitute 100% session availability).

### Logistic Regression Results

**FIGURE 2:**
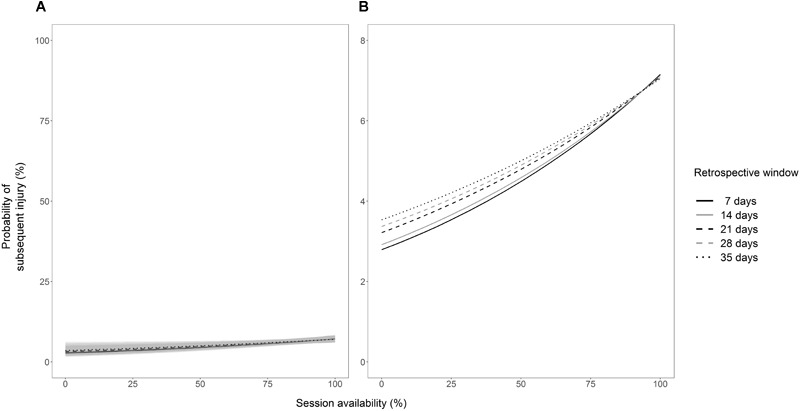
Subsequent injury probabilities estimated from statistically significant logistic regression models for session availability in the prior 7, 14, 21, 28, and 35 days. **(A,B)** Illustrate these data with the *y*-axis set between 0–100% and 0–8%, respectively, to highlight the importance of perspective when interpreting estimated injury probabilities. The gray shaded area indicates the 95% confidence intervals for the estimated injury probabilities.

**FIGURE 3:**
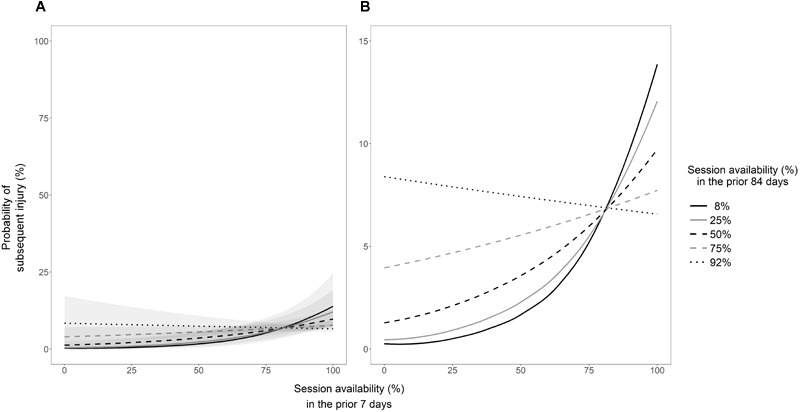
The interaction of session availability in the prior 7 days by session availability in the prior 84 days. The probability of subsequent injury was estimated at every possible value for session availability in the prior 7 days and fixed values for session availability in the prior 84 days. A value of 8% represents the minimum possible value for session availability in the prior 84 days when session availability in the prior 7 days equals 100%. Additionally, a value of 92% represents the maximum possible value for session availability in the prior 84 days when session availability in the prior 7 days equals 0%. The remaining values for session availability in the prior 84 days were chosen arbitrarily. **(A,B)** Illustrate these data with the *y*-axis set between 0–100% and 0–15%, respectively, to highlight the importance of perspective when interpreting estimated injury probabilities. The gray shaded area indicates the 95% confidence intervals for the estimated injury probabilities.

Out of the variables examined, session availability in the prior 7 days had the largest impact on the risk of subsequent injury. The effect, however, was small, with a 1% increase in session availability increasing the odds of injury by a factor of 1.0099 (95% CIs, 1.0036 to 1.0163). To give this context, in a 7 day window during which three sessions were possible, completing all three sessions (100% session availability), as opposed to two sessions (66.6% session availability), increased the odds of injury in the subsequent week 1.4 fold (95% CIs, 1.1 to 1.7). On a probability scale, however, the risk of subsequent injury was increased by 1.9%. The coefficients and 95% CIs (expressed as odds ratios) for each individual model can be found in Table 1. The estimated injury probabilities for every value of each statistically significant variable can be found in Figure 2. A statistically significant interaction was observed for session availability in the prior 14 days by games played in the prior season (coefficient = 0.9988, 95% CIs, 0.9978 to 0.9998). There were no other significant interactions of all session availability variables by age and by games played in the prior season (Supplementary Material S6). A statistically significant interaction of session availability in the prior 7 days by session availability in the prior 84 days was observed. The coefficient of this interaction (expressed as an odds ratio) was 0.9994 (95% CIs, 0.9991 to 0.9998). A visual representation of this interaction can be found in Figure 3.

**Table 1:** The results of individual logistic regression models built using each variable.

Variable		Coefficient (95% CIs)
Session availability (%) in the prior	7 days	1.0099 (1.0036 to 1.0163)^∗^
	14 days	1.0094 (1.0028 to 1.0161)^∗^
	21 days	1.0083 (1.0017 to 1.0150)^∗^
	28 days	1.0079 (1.0011 to 1.0146)^∗^
	35 days	1.0073 (1.0005 to 1.0141)^∗^
	42 days	1.0064 (0.9997 to 1.0132)
	49 days	1.0070 (1.0000 to 1.0139)
	56 days	1.0068 (0.9998 to 1.0138)
	63 days	1.0067 (0.9996 to 1.0138)
	70 days	1.0055 (0.9985 to 1.0125)
	77 days	1.0047 (0.9978 to 1.0117)
	84 days	1.0048 (0.9977 to 1.0119)
Age (years)		1.0300 (0.9900 to 1.0800)
Stature (cm)		1.0200 (1.0000 to 1.0400)
Mass (kg)		1.0100 (1.0000 to 1.0300)
Playing experience (years)		1.0200 (0.9800 to 1.0700)

Position	Back	Reference

	Forward	1.0800 (0.7400 to 1.5700)
	Ruck	1.0130 (0.5788 to 1.7531)
	Midfield	0.6700 (0.4400 to 1.0200)
Number of games played in the prior season		1.0001 (0.9813 to 1.0192)

*The coefficients are expressed as odds ratios and can be interpreted as the factor by which a one unit increase in the relevant variable affects the odds of a subsequent injury occurring. An odds ratio of less than 1 indicates the factor by which the odds of a subsequent injury occurring are decreased with a one unit increase in the predictor variable (e.g., a 1% increase in session availability). Conversely, an odds ratio of greater than 1 indicates the factor by which the odds of a subsequent injury occurring are increased with a one unit increase in the predictor variable. 95% CIs, 95% confidence intervals. ^∗^Indicates 95% CIs that do not include a value of 1.0000.*

## Discussion

The main findings of this study were that (1) greater session availability in the prior 7, 14, 21, 28, and 35 days increased the risk of injury and (2) lesser session availability in the prior 84 days increased the risk of injury only when coupled with greater session availability in the prior 7 days. In this investigation, session availability was used as a surrogate marker of the frequency/severity of prior injuries. Prior injury has been associated with an increased risk of subsequent injury (Orchard, 2001; Finch et al., 2017) and it could be argued that low levels of availability, as a surrogate marker of prior injury, may also be associated with an increased risk of injury (Windt et al., 2016; Finch et al., 2017). One study, conducted in elite rugby league, found that missing 10 training sessions over the pre-season period increased the odds of sustaining an injury during the season by 1.2 times (Windt et al., 2016). In the current study, missing the equivalent number of sessions over the prior 84 days equates to (on average) a 32% decrease in session availability. The current data suggest that a 32% decrease in session availability over the prior 84 days (when coupled with 100% availability in the prior 7 days) increases the odds of injury by approximately 1.4 times. It should be noted, however, that session availability in prior research (Windt et al., 2016) was not exclusive to injury and also included sessions missed due to illness, load management, and other reasons. The authors of the study (Windt et al., 2016) suggest that increased participation levels may decrease the risk of injury by allowing athletes to accumulate higher training loads and develop greater levels of strength and aerobic capacity, which are likely to have protective benefits (Windt et al., 2016). Alternatively, it is suggested that increased participation levels may simply identify individual athletes that are robust and already less susceptible to injury (Windt et al., 2016).

The hypothesis that greater session availability may be indicative of robustness, or a lesser susceptibility to injury, is partially supported by the findings of the current study. A significant interaction of session availability in the prior 14 days by the number of games played in the prior season was observed (Supplementary Material S6). Whilst this interaction was weak, it suggests that athletes who played more games in the prior season may be more robust and less susceptible to acute increases in session availability. The results of the univariable models (Table 1), however, are in contrast with previous research (Windt et al., 2016). These data suggest that athletes who trained and played more in the prior 7, 14, 21, 28, and 35 days were at an increased risk of subsequent injury when compared to athletes who missed more training sessions and matches due to injury. Of the individual session availability variables, availability in the prior 7 days had the largest (albeit a small) impact on injury risk, with an increase in availability increasing the probability of injury by up to 4.4% (Figure 2). In light of this, it could be hypothesized that greater acute availability (i.e., prior 7 days) equates to higher acute training loads, which have been associated with an increased risk of injury (Gabbett, 2016). However, as previously mentioned, it could also be hypothesized that greater availability for prolonged periods (i.e., prior 84 days) equates to higher chronic training loads, which have been associated with a decreased risk of injury (Gabbett, 2016). When examined in isolation, greater session availability in the prior 84 days did not decrease the risk of subsequent injury. However, based on the interaction of session availability in the prior 84 days by session availability in the prior 7 days, low levels of chronic availability coupled with high levels of acute availability increased the risk of injury by up to 14.1% (Figure 3).

The relationship illustrated in Figure 3 supports the evidence relating to acute and chronic training loads and their impact on the risk of injury (Carey et al., 2016; Gabbett, 2016; Murray et al., 2016). Summarizing the relationship between acute and chronic training loads using a ratio is an approach that has been widely investigated and adopted by practitioners (Carey et al., 2016, 2018a; Gabbett, 2016; Murray et al., 2016). Prior research suggests that acute to chronic training load ratios of greater than 1.5 increase the risk of injury and should be considered a “danger zone” (Gabbett, 2016). The physiological mechanisms by which training load influences the risk of injury are still not entirely understood (Windt and Gabbett, 2017). It has been suggested that greater training loads simply increase an athlete’s level of exposure to the possibility of an inciting event, therefore increasing their risk of injury (Windt and Gabbett, 2017). The same relationship may exist in the current data, whereby greater session availability increases an athlete’s level of exposure, which in turn increases their risk of injury. However, as previously mentioned greater chronic training loads have been associated with a decreased risk of injury (Gabbett, 2016). It has been suggested that greater chronic training loads allow athletes to develop greater levels of fitness (i.e., strength, aerobic capacity, anaerobic capacity) and decrease their susceptibility to fatigue, thereby reducing their risk of injury (Windt et al., 2017). Equally, greater acute training loads are thought to induce fatigue and increase the risk of injury, through diminished neuromuscular control and tissue capacity (Windt and Gabbett, 2017). Accordingly, maintaining an appropriate training load ratio is thought to maximize the protective benefits of greater chronic training loads while minimizing the harmful effects of greater acute training loads (Gabbett, 2016). However, the validity of this idea has been questioned, as individual athletes are likely to tolerate given training load ratios differently (Buchheit, 2017). An individual approach that accounts for differing physical qualities (i.e., age, injury history, fitness levels) may be important when investigating the impact of training loads on injury risk (Buchheit, 2017).

Despite the hypotheses regarding training loads and injury risk, the relationship between session availability and training load is yet to be investigated. However, altered or interrupted training loads following an injury may provide insight into the potential mechanisms by which prior injury (and consequently session availability) impacts the risk of subsequent injury. However, it should be noted that in the current data, summarizing acute and chronic availability using a ratio leads to a loss of information, likely due to the aforementioned limitations. For example, when acute availability (prior 7 days) equals 12% and chronic availability (prior 84 days) equals 8%, the ratio between these two values is equal to 1.5. The probability of injury, however, is only 0.3%. In contrast, when acute availability and chronic availability both equal to 92% and the ratio is equal to 1.0, the probability of injury is approximately 22 times higher (Figure 3). Whilst these data are interpolated and subject to error, this example highlights the importance of examining the statistical interaction between multiple variables, as opposed to summarizing their relationship using a ratio. Practically, acute to chronic training load ratios can provide valuable information regarding the progression of training but should not be used in isolation when attempting to identify injury risk.

Prior research has typically investigated the direct impact of training loads on the risk of injury (Gabbett, 2016; Windt and Gabbett, 2017). It has been suggested, however, that prior injury is likely to impact training loads via the amount of training/competition missed during an injury layoff (Windt et al., 2016; Finch et al., 2017). Given the current data, it could be argued that session availability, as a surrogate marker of prior injury, may also be a surrogate (and potentially more easily accessible) marker of training load. However, in the current study, injured athletes that were not available for field/skills training or matches still likely completed rehabilitation sessions. The training loads that athletes are exposed to during rehabilitation sessions are dependent on the stage of rehab as well as the pathology. For example, an athlete with an upper limb injury may still complete the same amount of running as the rest of the team but is unable to participate in some football specific drills. Conversely, an athlete with a lower limb injury may not participate in any running or football specific drills. Whilst in both cases the athletes are considered unavailable, they may be exposed to very different training loads. Further research investigating the impact of session availability and specific pathologies on both external and internal training loads may provide greater insight into the relationship between session availability and injury risk. However, the ability to examine the impact of specific pathologies is a current limitation of injury research (van Dyk et al., 2017).

The current study has a number of potential limitations. Firstly, any training sessions or matches that were missed due to reasons other than an injury (i.e., illness, personal reasons and other commitments) were censored from the data and this may have masked the impact these factors could have had on the risk of subsequent injury. This decision was made in an attempt to isolate the impact of prior injury on session availability and subsequently future injury risk. The authors acknowledge that the analysis conducted in the current study could have been undertaken a number of different ways (e.g., to include training sessions and matches that were missed due to illness, personal reasons and other commitments). We are confident, however, that due to the small proportion of training sessions and matches that were missed due to non-injury related circumstances (Supplementary Material S1), the findings of the current study would not have been influenced by this decision. Secondly, an injury was defined as any physical complaint (excluding illness) that resulted in at least one missed/modified training session or match. Any injury was considered when calculating session availability (Finch et al., 2017). However, only non-contact lower limb injuries were considered when identifying prospective injuries (Colby et al., 2018). It is important to note that the application of different definitions when calculating session availability and identifying subsequent injury risk may produce different results from those observed in the current study. Lastly, from a statistical perspective, logistic regression assumes independence between observations. In reality, rolling calculations of session availability are not true independent observations, but repeated measures from individual athletes. Accounting for individual athletes may produce different results; however, previous research has observed no difference when modeling training load data as both individual observations and repeated measures (Carey et al., 2018b).

When attempting to identify injury risk on an individual level, a complex approach should be taken (Ruddy et al., 2017). A complex approach can help better identify and account for the multifaceted, non-linear interactions that occur between multiple risk factors (Bittencourt et al., 2016). However, research identifying factors that are associated with injury risk is vital in helping to inform and implement a complex systems approach. Whilst it is non-modifiable, session availability is likely to moderate the impact of other factors on the risk of injury and should be considered when implementing a complex approach to identify injury risk. However, the results of the current study should be interpreted with caution. The largest increase in the probability of subsequent injury estimated from the univariable models (Figure 2) and the interaction model (Figure 3) was 4.4 and 14.1%, respectively. Both Figure 2, 3 have been visualized with the y-axes set at different intervals to highlight the importance of perspective when interpreting estimated injury probabilities. A statistically significant increase in the likelihood of an outcome occurring may not necessarily be considered practically important by a coach or clinician (Nielsen et al., 2017). It is the responsibility of coaches and clinician to understand and interpret data in a context-specific manner when making decisions (Nielsen et al., 2017; Colby et al., 2018). Equally, it is the responsibility of researchers to conduct meaningful analyses and ensure that data are not misinterpreted.

## Conclusion

Acute increases in session availability, when examined in isolation, were associated with an increased risk of subsequent injury. Lesser session availability over prolonged periods were associated with an increased risk of subsequent injury, only when coupled with acute increases in session availability. Practically, coaches and clinicians can use this information to help plan and guide the progression of training, particularly for athletes that are returning from long periods of injury. The impact of session availability and specific pathologies on external training loads is yet to be investigated, but could possibly provide further insight into the mechanisms responsible for the findings of the current study. The aforementioned considerations aside, session availability may be an informative marker of the impact of prior injury on subsequent injury risk and should be considered in concert with other variables when attempting to identify injury risk on an individual level.

## Ethics Statement

This study was approved by the Australian Catholic University Human Research Ethics Committee (approval number: 2018-26WN).

## Author Contributions

JR, SP, SC, and DO contributed to the design of the study. JR, SP, and SC contributed to the collection of the data. JR and NM performed the data analysis. JR, NM, and DC performed the statistical analysis. JR drafted the manuscript. SP, NM, SC, RT, MW, DC, and DO contributed to the manuscript.

## Conflict of Interest Statement

The authors declare that the research was conducted in the absence of any commercial or financial relationships that could be construed as a potential conflict of interest.

## Supplementary Material

MATERIAL S1 The number and proportion of training sessions and matches fully completed and missed/modified due to various reasons during the 2015, 2016 and 2017 Australian Football League seasons, including both the pre-season and in-season periods. An injury is defined as any physical complaint (excluding illness) that resulted in at least one missed/modified training session or match.

MATERIAL S2 A visual depiction of the data analysis steps taken for the current study. In this figure, Week 8 is used as an example. However, the reader should note that these step were applied for each individual athlete, for every week (i.e., Monday to Sunday) during the in-season periods. For every Monday during the in-season periods, the number of training sessions and matches that were missed/modified due to injury in the prior 7, 14, 21, 28, 35, 42, 49, 56, 63, 70, 77, and 84 days was determined for each athlete. The number of full training sessions and matches that each athlete could have conceivably completed (if injury free) was also determined for the same retrospective windows. Session availability (%) for each retrospective window was then determined as the number of training sessions and matches fully completed (injury free) relative to the number of training sessions and matches possible for each athlete. Non-contact lower limb injuries that occurred during the following 7 days (inclusive of the index day, i.e., Monday to Sunday) were identified as subsequent injuries. Any type of injury, given it resulted in at least one missed/modified training session or match, was considered when calculating session availability. Only non-contact lower limb injuries were considered as subsequent injuries. Any weeks during which an athlete did not train or play (i.e., was not exposed to the risk of subsequent injury) were not considered when identifying subsequent injury risk, but were still considered when calculating session availability.

MATERIAL S3 Logistic regression equations.

MATERIAL S4 The correlation between each individual session availability variable. Session availability is determined as the number of training sessions and matches fully completed relative to the number of training sessions and matches in each of the retrospective windows.

MATERIAL S5 The incidence and prevalence of injuries by location for the 2015, 2016 and 2017 Australian Football League seasons, including the pre-season and in-season periods. An injury is defined as any physical complaint (excluding illness) that resulted in at least one missed/modified training session or match.

MATERIAL S6 The logistic regression results of the individual interactions between session availability in each retrospective window and age and games played in the prior season. The coefficients are expressed as odds ratios (OR).
