# Supplementary material for: Session Availability as a Result of Prior Injury Impacts the Risk of Subsequent Non-contact Lower Limb Injury in Elite Male Australian Footballers
Source: Front Physiol. 2019 Jun 14;10:737. doi: 10.3389/fphys.2019.00737 (PMC6593276; doi:10.3389/fphys.2019.00737)
Supplement: MATERIAL S1 — The number and proportion of training sessions and matches fully completed and missed/modified due to various reasons during the 2015, 2016 and 2017 Australian Football League seasons, including both the pre-season and in-season periods. An injury is defined as any physical complaint (excluding illness) that resulted in at least one missed/modified training session or match. [file Data_Sheet_1.zip › Supplementary Material 1.docx]

| Participation status | Reason | Number of training sessions and matches | Proportion of total training sessions and matches (%) |
| --- | --- | --- | --- |
| Fully completed |  | 10339 | 74.8 |
| Missed or modified due to | Injury | 2925 | 21.2 |
|  | Illness | 59 | 0.4 |
|  | Club/personal commitments | 188 | 1.4 |
|  | Suspension | 21 | 0.2 |
|  | Resting/management | 290 | 2.1 |

**Supplementary Material 1.** The number and proportion of training sessions and matches fully completed and missed/modified due to various reasons during the 2015, 2016 and 2017 Australian Football League seasons, including both the pre-season and in-season periods. An injury is defined as any physical complaint (excluding illness) that resulted in at least one missed/modified training session or match.
