# Supplementary material for: Session Availability as a Result of Prior Injury Impacts the Risk of Subsequent Non-contact Lower Limb Injury in Elite Male Australian Footballers
Source: Front Physiol. 2019 Jun 14;10:737. doi: 10.3389/fphys.2019.00737 (PMC6593276; doi:10.3389/fphys.2019.00737)
Supplement: MATERIAL S1 — The number and proportion of training sessions and matches fully completed and missed/modified due to various reasons during the 2015, 2016 and 2017 Australian Football League seasons, including both the pre-season and in-season periods. An injury is defined as any physical complaint (excluding illness) that resulted in at least one missed/modified training session or match. [file Data_Sheet_1.zip › Supplementary Material 3.docx]

**Supplementary Material 3.** Logistic regression equations

Univariable equation:

$$Probability of injury= \frac{1}{1+exp(-(B_{0}+B_{1}X_{1}))}$$

Where B_0_ equals the sample intercept, B_1_ equals the coefficient of the independent variable and X_1_ equals the value of the independent variable.

This equation was used to estimate the probability of injury given the following individual variables:

- Session availability (%) in the prior 7 days
- Session availability (%) in the prior 14 days
- Session availability (%) in the prior 21 days
- Session availability (%) in the prior 28 days
- Session availability (%) in the prior 35 days
- Session availability (%) in the prior 42 days
- Session availability (%) in the prior 49 days
- Session availability (%) in the prior 56 days
- Session availability (%) in the prior 63 days
- Session availability (%) in the prior 70 days
- Session availability (%) in the prior 77 days
- Session availability (%) in the prior 84 days
- Age
- Stature
- Mass
- Playing experience
- Position
- Number of games played in the prior season

Interaction equation:

$$Probability of injury= \frac{1}{1+exp(-(B_{0}+B_{1}X_{1}+B_{2}X_{2}+B_{3}\left( X_{1}X_{2} \right)))}$$

Where B_0_ equals the sample intercept, B_1_ equals the coefficient of the first independent variable, X_1_ equals the value of the first independent variable, B_2_ equals the coefficient of the second independent variable, X_2_ equals the value of the second independent variable, and B_3_ equals the interaction coefficient.

This equation was used to estimate the probability of injury given the following interactions:

- Session availability (%) in the prior 7 days × session availability (%) in the prior 84 days
- Age × session availability (%) in the prior 7 days
- Age × session availability (%) in the prior 14 days
- Age × session availability (%) in the prior 21 days
- Age × session availability (%) in the prior 28 days
- Age × session availability (%) in the prior 35 days
- Age × session availability (%) in the prior 42 days
- Age × session availability (%) in the prior 49 days
- Age × session availability (%) in the prior 56 days
- Age × session availability (%) in the prior 63 days
- Age × session availability (%) in the prior 70 days
- Age × session availability (%) in the prior 77 days
- Age × session availability (%) in the prior 84 days
- Number of games played in the prior season × session availability (%) in the prior 7 days
- Number of games played in the prior season × session availability (%) in the prior 14 days
- Number of games played in the prior season × session availability (%) in the prior 21 days
- Number of games played in the prior season × session availability (%) in the prior 28 days
- Number of games played in the prior season × session availability (%) in the prior 35 days
- Number of games played in the prior season × session availability (%) in the prior 42 days
- Number of games played in the prior season × session availability (%) in the prior 49 days
- Number of games played in the prior season × session availability (%) in the prior 56 days
- Number of games played in the prior season × session availability (%) in the prior 63 days
- Number of games played in the prior season × session availability (%) in the prior 70 days
- Number of games played in the prior season × session availability (%) in the prior 77 days
- Number of games played in the prior season × session availability (%) in the prior 84 days
