# Supplementary material for: Session Availability as a Result of Prior Injury Impacts the Risk of Subsequent Non-contact Lower Limb Injury in Elite Male Australian Footballers
Source: Front Physiol. 2019 Jun 14;10:737. doi: 10.3389/fphys.2019.00737 (PMC6593276; doi:10.3389/fphys.2019.00737)
Supplement: MATERIAL S1 — The number and proportion of training sessions and matches fully completed and missed/modified due to various reasons during the 2015, 2016 and 2017 Australian Football League seasons, including both the pre-season and in-season periods. An injury is defined as any physical complaint (excluding illness) that resulted in at least one missed/modified training session or match. [file Data_Sheet_1.zip › Supplementary Material 4.docx]

| Retrospective window | 7 days | 14 days | 21 days | 28 days | 35 days | 42 days | 49 days | 56 days | 63 days | 70 days | 77 days | 84 days |
| --- | --- | --- | --- | --- | --- | --- | --- | --- | --- | --- | --- | --- |
| 7 days | - | 0.90 | 0.83 | 0.77 | 0.72 | 0.68 | 0.65 | 0.62 | 0.60 | 0.58 | 0.56 | 0.54 |
| 14 days | 0.90 | - | 0.95 | 0.90 | 0.84 | 0.80 | 0.76 | 0.73 | 0.70 | 0.67 | 0.65 | 0.63 |
| 21 days | 0.83 | 0.95 | - | 0.97 | 0.92 | 0.88 | 0.84 | 0.80 | 0.77 | 0.74 | 0.72 | 0.70 |
| 28 days | 0.77 | 0.90 | 0.97 | - | 0.98 | 0.94 | 0.90 | 0.87 | 0.83 | 0.80 | 0.78 | 0.76 |
| 35 days | 0.72 | 0.84 | 0.92 | 0.98 | - | 0.98 | 0.95 | 0.92 | 0.89 | 0.86 | 0.83 | 0.81 |
| 42 days | 0.68 | 0.80 | 0.88 | 0.94 | 0.98 | - | 0.99 | 0.96 | 0.93 | 0.90 | 0.88 | 0.85 |
| 49 days | 0.65 | 0.76 | 0.84 | 0.90 | 0.95 | 0.99 | - | 0.99 | 0.97 | 0.94 | 0.91 | 0.89 |
| 56 days | 0.62 | 0.73 | 0.80 | 0.87 | 0.92 | 0.96 | 0.99 | - | 0.99 | 0.97 | 0.95 | 0.93 |
| 63 days | 0.60 | 0.70 | 0.77 | 0.83 | 0.89 | 0.93 | 0.97 | 0.99 | - | 0.99 | 0.97 | 0.95 |
| 70 days | 0.58 | 0.67 | 0.74 | 0.80 | 0.86 | 0.90 | 0.94 | 0.97 | 0.99 | - | 0.99 | 0.98 |
| 77 days | 0.56 | 0.65 | 0.72 | 0.78 | 0.83 | 0.88 | 0.91 | 0.95 | 0.97 | 0.99 | - | 0.99 |
| 84 days | 0.54 | 0.63 | 0.70 | 0.76 | 0.81 | 0.85 | 0.89 | 0.93 | 0.95 | 0.98 | 0.99 | - |

**Supplementary Material 4.** The correlation between each individual session availability variable. Session availability is determined as the number of training sessions and matches fully completed relative to the number of training sessions and matches in each of the retrospective windows.
