# Supplementary material for: Session Availability as a Result of Prior Injury Impacts the Risk of Subsequent Non-contact Lower Limb Injury in Elite Male Australian Footballers
Source: Front Physiol. 2019 Jun 14;10:737. doi: 10.3389/fphys.2019.00737 (PMC6593276; doi:10.3389/fphys.2019.00737)
Supplement: MATERIAL S1 — The number and proportion of training sessions and matches fully completed and missed/modified due to various reasons during the 2015, 2016 and 2017 Australian Football League seasons, including both the pre-season and in-season periods. An injury is defined as any physical complaint (excluding illness) that resulted in at least one missed/modified training session or match. [file Data_Sheet_1.zip › Supplementary Material 5.docx]

**Supplementary Material 5.** The incidence and prevalence of injuries by location for the 2015, 2016 and 2017 Australian Football League seasons, including the pre-season and in-season periods. An injury is defined as any physical complaint (excluding illness) that resulted in at least one missed/modified training session or match.

| Season | Mechanism | Location | Total number of injuries | Median number of training sessions impacted (IQR) | Median number of matches impacted (IQR) |
| --- | --- | --- | --- | --- | --- |
| 2015 | Contact | Lower limb | 46 | 1 (2) | 0 (1) |
|  |  | Trunk/head | 30 | 2 (2) | 1 (2) |
|  |  | Upper limb | 9 | 3 (4) | 0 (3) |
|  | Non-contact | Lower limb | 138 | 1 (3) | 0 (1) |
|  |  | Trunk/head | 17 | 2 (1) | 0 (2) |
|  |  | Upper limb | 3 | 2 (3) | 0 (2) |
| 2016 | Contact | Lower limb | 46 | 1 (1) | 0 (1) |
|  |  | Trunk/head | 24 | 2 (3) | 1 (2) |
|  |  | Upper limb | 23 | 1 (2) | 0 (1) |
|  | Non-contact | Lower limb | 107 | 2 (4) | 0 (2) |
|  |  | Trunk/head | 14 | 1 (1) | 0 (1) |
|  |  | Upper limb | 2 | 5 (1) | 2 (5) |
| 2017 | Contact | Lower limb | 51 | 2 (3) | 0 (2) |
|  |  | Trunk/head | 19 | 2 (2) | 0 (2) |
|  |  | Upper limb | 17 | 5 (11) | 0 (5) |
|  | Non-contact | Lower limb | 107 | 2 (5) | 0 (2) |
|  |  | Trunk/head | 12 | 2 (2) | 0 (2) |
|  |  | Upper limb | 1 | 3 (0) | 1 (3) |

IQR; interquartile range
