# Supplementary material for: Session Availability as a Result of Prior Injury Impacts the Risk of Subsequent Non-contact Lower Limb Injury in Elite Male Australian Footballers
Source: Front Physiol. 2019 Jun 14;10:737. doi: 10.3389/fphys.2019.00737 (PMC6593276; doi:10.3389/fphys.2019.00737)
Supplement: MATERIAL S1 — The number and proportion of training sessions and matches fully completed and missed/modified due to various reasons during the 2015, 2016 and 2017 Australian Football League seasons, including both the pre-season and in-season periods. An injury is defined as any physical complaint (excluding illness) that resulted in at least one missed/modified training session or match. [file Data_Sheet_1.zip › Supplementary Material 6.docx]

**Supplementary Material 6.** The logistic regression results of the individual interactions between session availability in each retrospective window and age and games played in the prior season. The coefficients are expressed as odds ratios (OR).

|  | Age | Games played in the prior season |
| --- | --- | --- |
| Retrospective window | OR (95% CIs) | OR (95% CIs) |
| 7 days | 0.9999 (0.9981 to 1.0018) | 0.9991 (0.9982 to 1.0000) |
| 14 days | 0.9994 (0.9975 to 1.0014) | 0.9988 (0.9978 to 0.9998)* |
| 21 days | 0.9997 (0.9977 to 1.0016) | 0.9992 (0.9983 to 1.0001) |
| 28 days | 0.9997 (0.9978 to 1.0017) | 0.9995 (0.9987 to 1.0003) |
| 35 days | 1.0000 (0.9980 to 1.0020) | 0.9997 (0.9989 to 1.0005) |
| 42 days | 1.0001 (0.9981 to 1.0021) | 0.9997 (0.9989 to 1.0005) |
| 49 days | 1.0003 (0.9982 to 1.0024) | 0.9997 (0.9989 to 1.0005) |
| 56 days | 1.0000 (0.9979 to 1.0021) | 0.9997 (0.9988 to 1.0005) |
| 63 days | 0.9999 (0.9978 to 1.0021) | 0.9997 (0.9988 to 1.0005) |
| 70 days | 0.9997 (0.9976 to 1.0018) | 0.9997 (0.9988 to 1.0005) |
| 77 days | 0.9994 (0.9973 to 1.0015) | 0.9997 (0.9988 to 1.0005) |
| 84 days | 0.9993 (0.9972 to 1.0014) | 0.9996 (0.9988 to 1.0005) |

95% CIs; 95% confidence intervals

*indicates 95% CIs that do not include a value of 1.0000
