# Supplementary figures and images for: Session Availability as a Result of Prior Injury Impacts the Risk of Subsequent Non-contact Lower Limb Injury in Elite Male Australian Footballers
Source: Front Physiol. 2019 Jun 14;10:737. doi: 10.3389/fphys.2019.00737 (PMC6593276; doi:10.3389/fphys.2019.00737)

## In-season period

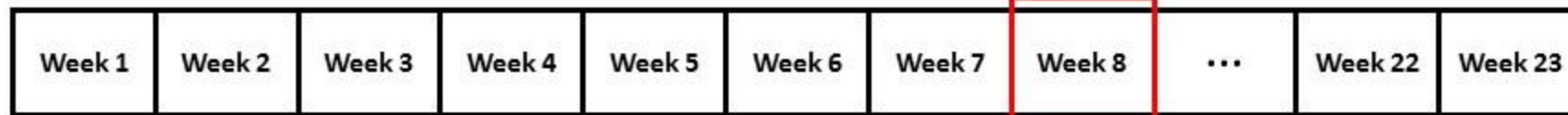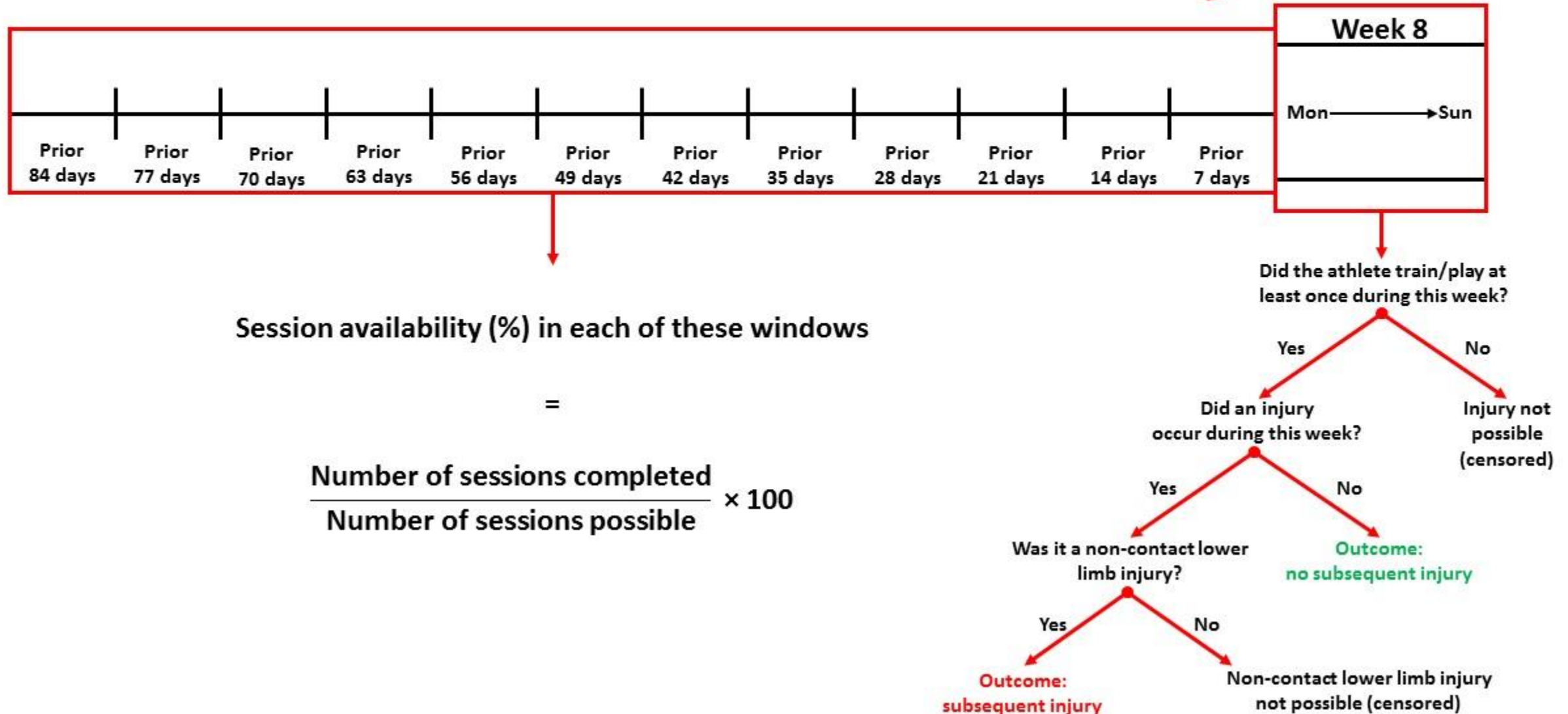

Supplement: MATERIAL S1 — The number and proportion of training sessions and matches fully completed and missed/modified due to various reasons during the 2015, 2016 and 2017 Australian Football League seasons, including both the pre-season and in-season periods. An injury is defined as any physical complaint (excluding illness) that resulted in at least one missed/modified training session or match. [file Data_Sheet_1.zip › Supplementary Material 2.pdf]
